# Deoxynucleoside supplementation ameliorates the disease associated phenotypes in a zebrafish model of *RRM2B* mtDNA depletion syndrome

**DOI:** 10.1093/hmg/ddaf047

**Published:** 2025-04-11

**Authors:** Benjamin Munro, Declan Hines, Juliane S Mueller, Rita Horvath

**Affiliations:** John Van Geest Centre for Brain Repair, Department of Clinical Neurosciences, University of Cambridge, Robinson Way, Cambridge CB2 0PY, United Kingdom; School of Clinical Medicine, University of Cambridge, Hills Road, Cambridge CB2 0SP, United Kingdom; John Van Geest Centre for Brain Repair, Department of Clinical Neurosciences, University of Cambridge, Robinson Way, Cambridge CB2 0PY, United Kingdom; Dubowitz Neuromuscular Centre, Great Ormond Street Hospital for Children NHS Foundation Trust, UCL, 30 Guilford Street, London WC1N 1EH, United Kingdom; John Van Geest Centre for Brain Repair, Department of Clinical Neurosciences, University of Cambridge, Robinson Way, Cambridge CB2 0PY, United Kingdom

**Keywords:** MDDS, Mitochondrial DNA depletion syndromes, zebrafish, Deoxynucleoside supplementation, RRM2B

## Abstract

Mitochondrial DNA (mtDNA) depletion syndromes (MDDS) are rare, clinically heterogeneous mitochondrial disorders resulting from nuclear variants in genes of the mitochondrial DNA replication or maintenance machinery. Supplementation with pyrimidine deoxynucleosides have been beneficial in patients and mice with *TK2*-related MDDS, however, it has not been systematically explored in other forms of MDDS. To investigate the effect of deoxynucleoside supplementation in mitigating the disease in mitochondrial DNA depletion due to pathogenic *RRM2B* variants, we generated a novel zebrafish knock-out model of this disease and studied the effect of different combinations of deoxynucleosides. Zebrafish larvae carrying a homozygous nonsense mutation in *rrm2b* present with impaired movement, reduced mtDNA copy number and elevated lactate. Supplementation with different combination of deoxynucleosides was performed, resulting in increased mtDNA copy numbers when supplemented with the two purine deoxynucleosides (dGuo and dAdo), while other combinations had no effect or even further compromised mtDNA copy number in zebrafish. In parallel with increased mtDNA copy number, we detected improved movement and reduction of lactate in the *rrm2b^−/−^* fish, confirming the beneficial effect of deoxynucleosides on the whole organism. This treatment did not result in any deleterious effect in wild type and heterozygous fish. Our data suggest that supplementation with deoxynucleosides may be beneficial and should be further investigated in *RRM2B*-related disease, adding to the growing evidence that it is a valid therapeutic approach which can be trialled for treating a wider range of genetic forms of MDDS.

## Introduction

Mitochondrial DNA (mtDNA) depletion syndromes (MDDS) are a group of rare, clinically heterogeneous, autosomal recessive disorders resulting from defects in mitochondrial DNA replication or maintenance. The primary hallmark of MDDS is reduced level of mtDNA (mtDNA depletion), which is sometimes associated with multiple mtDNA deletions or point mutations, in post-mitotic, high energy demand tissues. Disease presentations of recessive MDDS range from severe infantile or childhood-onset intractable epilepsy, liver failure and psychomotor regression (Alpers Syndrome) to infantile liver failure, mitochondrial myopathy or encephalomyopathy [1–3]. Depletion of mtDNA can be attributed to defects in the mtDNA replication machinery due to pathogenic variants in genes including *POLG*, *TFAM, TWNK* and *SSBP1* [4–8] and in those associated with mitochondrial nucleotide pool maintenance, including *TK2, DGUOK, TYMP, RRM2B* and *GUK1* [9–13].

*RRM2B* encodes the protein RRM2B (p53R2), which in post-mitotic cells, acts as the small subunit of the heterotetrametric cytosolic protein complex, Ribonucleotide Reductase (RNR), responsible for the reduction of nucleoside diphosphates (NDPs) to deoxynucleoside diphosphates (dNDPs), which is a key step in *de novo* dNTP synthesis [14]. Following subsequent reactions into dNTPs, these nucleotides can be used in mtDNA replication and repair. *RRM2B* is expressed exclusively in response to DNA damage or in post-mitotic cells when the RRM2 small sub-unit is actively degraded [15, 16]. Recessive pathogenic variants in *RRM2B* typically present in infants or young children, with severe tissue specific symptoms, often leading to premature death. Symptoms include neonatal hypotonia, neurological deterioration, seizures, severe lactic acidosis, sensorineural hearing loss and renal tubulopathy with an extreme tissue specific depletion of mtDNA [11, 17–19]. There is currently no approved treatment available for patients with pathogenic *RRM2B* variants, however, deoxynucleoside supplementation has been shown to be effective in reversing the disease symptoms of *TK2-*related MDDS in patients [20, 21], which built on the initial success of treating Tk2 deficient mice with pyrimidine deoxynucleosides [22–24].

Both *in vitro* and *in vivo* models of MDDS due to pathogenic variants in *POLG* [25–27]*, RRM2B* [28] and *DGUOK* [25, 29] have been used to test the efficacy of nucleotide supplementation and have had positive results, indicating deoxynucleoside therapy as a candidate for treating these other forms of MDDS. However, due to the heterogenous nature of these diseases, we cannot necessarily conclude that one treatment can be applied to patients with all different forms. Further studies are needed to determine whether deoxynucleoside supplementation is safe and effective in other forms of MDDS.

To investigate the effect of deoxynucleoside supplementation in mitigating the disease in *RRMB2* MDDS, we generated a novel zebrafish knock-out model. Transgenic *rrm2b* mutant fish recapitulate the hallmarks of the mtDNA depletion syndrome with impaired movement, decreased mtDNA copy number and elevated lactate levels. Our data show these phenotypes can be attenuated with supplementation of purine deoxynucleosides, adding to the growing evidence that deoxynucleoside supplementation is a valid therapeutic approach which can be trialled for treating a wider range of genetic forms of MDDS.

## Results

### Generating a zebrafish *rrm2b* knock-out model

**Figure 1:**
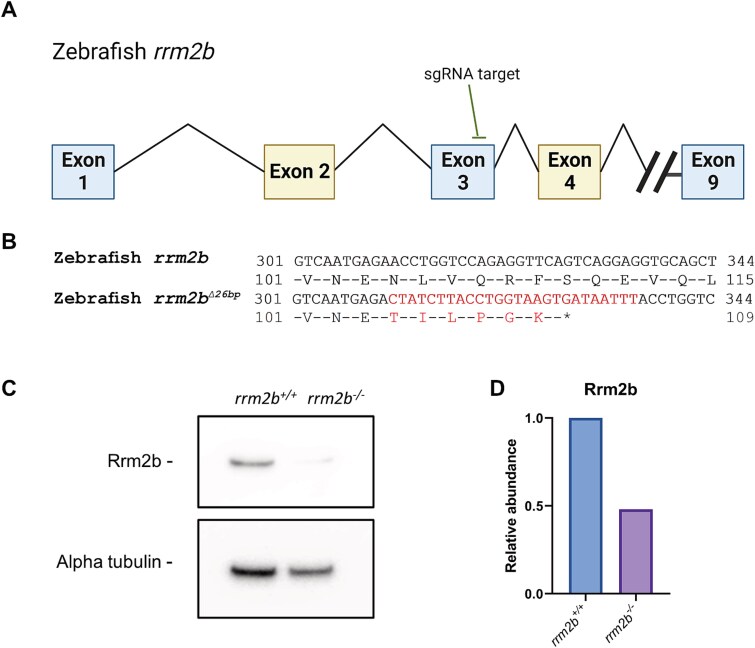
Mutagenesis of zebrafish *rrm2b* –. (A) Summary schematic depicting the *rrm2b* sgRNA target site on exon 3 of the zebrafish *rrm2b* gene. (B) DNA and corresponding peptide sequence of WT zebrafish *rrm2b* and mutant zebrafish *rrm2b^∆26bp^*. Red = mutation, ^*^ = premature stop codon. (C) Immunoblotting of Rrm2b and alpha tubulin in *rrm2b^+/+^* and *rrm2b^−/−^* in 5 dpf tail muscle*.* N = 10 larvae tails each. D. Quantification of Rrm2b immunoblotting relative to alpha tubulin.

To study the effects of deoxynucleoside supplementation *in vivo,* a zebrafish *rrm2b* knock-out model has been generated. Zebrafish have only one orthologue of human *RRM2B* (ENSDARG00000033367.7) which encodes a protein of 349 amino acids in length, sharing a 72.6% sequence identity with the human protein and with high amino acid sequence conservation across several model species (Supplementary Fig. 1). In order to generate an *rrm2b* knock-out model, we designed a sgRNA targeting exon 3 of zebrafish *rrm2b* (Fig. 1A). Following a CRISPR/Cas9 mutagenesis protocol [30], a zebrafish mutant with a 26-base pair (bp) insertion in exon 3 of *rrm2b* was developed (c.312_313ins CTATCTTACCTGGTAAGTGATAATTT), from here referred to as *rrm2b^−/−^* and *rrm2b^+/−^* for homozygous and heterozygous zebrafish for this mutation. This mutation is predicted to introduce a premature stop codon (Fig. 1B) and is shown to reduce Rrm2b protein in 5 dpf *rrm2b^−/−^* larvae (Fig. 1C).

### *The rrm2b^−/−^* zebrafish have impaired escape response, swim bladder inflation and lifespan

**Figure 2:**
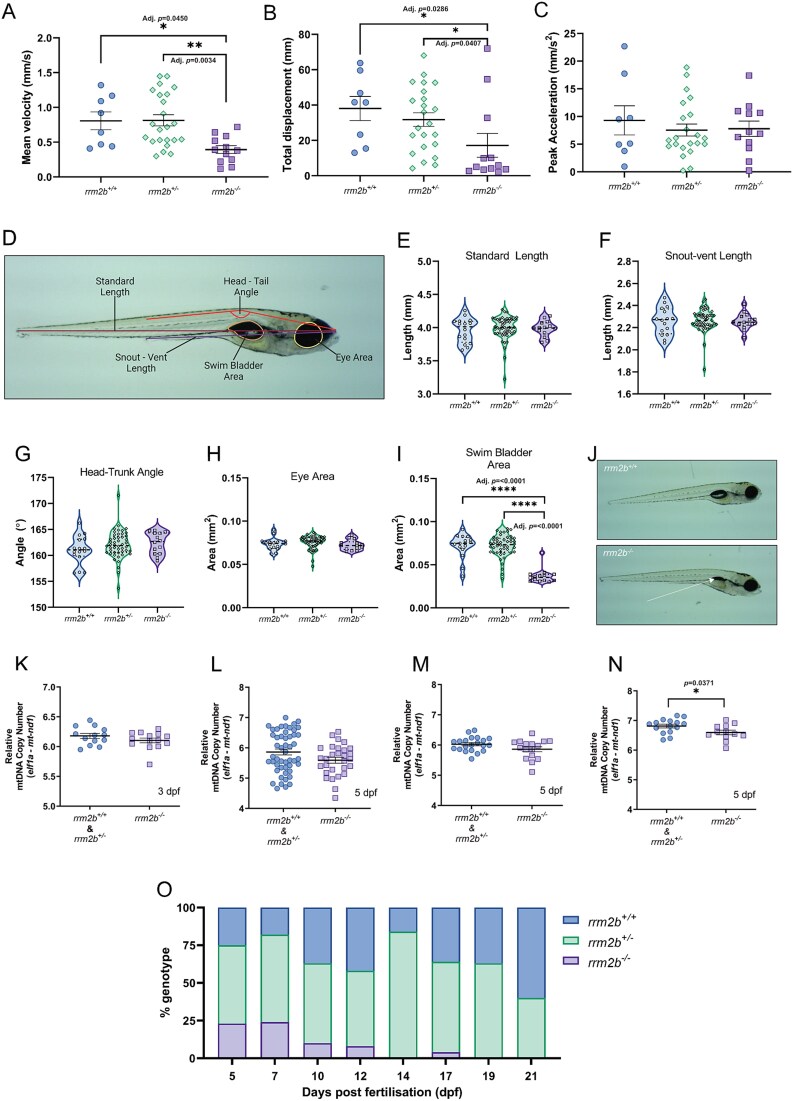
Characterisation of *rrm2b^−/−^* mutants—. (A) Mean velocity (mm/s) after touch stimulus of 2 dpf *rrm2b^+/+^* (*n* = 8), *rrm2b^+/−^* (*n* = 23) and *rrm2b^−/−^* larvae (*n* = 12). Results are shown as mean ± SEM (statistical significance was determined with a Kruskal-Wallis test, ^*^ = adj. *P* < 0.05, ^*^^*^ = adj. *P* < 0.01). (B) Total displacement (mm) after touch stimulus of 2 dpf *rrm2b^+/+^* (*n* = 8), *rrm2b^+/−^* (*n* = 23) and *rrm2b^−/−^* larvae (*n* = 12). Results are shown as mean ± SEM (statistical significance was determined with a Kruskal-Wallis test, ^*^ = adj. *P* < 0.05). (C) Peak acceleration (mm/s^2^) after touch stimulus of 2 dpf *rrm2b^+/+^* (*n* = 8), *rrm2b*^+/−^ (*n* = 22) and *rrm2b^−/−^* larvae (*n* = 12). Results are shown as mean ± SEM. (D) Representative image illustrating the morphological parameters used to assess zebrafish larval development. (E) Standard length (SL) measurements of 5 dpf r*rm2b^+/+^* (*n* = 16), *rrm2b^+/Δ26bp^* (*n* = 48) and *rrm2b^−/−^*(*n* = 14) larvae. Results are shown as mean ± SEM. (F) Snout-vent length (SVL) of 5 dpf *rrm2b^+/+^* (*n* = 16), *rrm2b^+/−^* (*n* = 48) and *rrm2b^−/−^* (*n* = 14) larvae. Results are shown as mean ± SEM. (G) Head-trunk angle (HTA) measurements of 5 dpf *rrm2b^+/+^* (*n* = 16), *rrm2b^+/−^* (*n* = 48) and *rrm2b^−/−^* (*n* = 14) larvae. Results are shown as mean ± SEM. (H) Eye area (EA) measurements of 5 dpf *rrm2b^+/+^* (*n* = 16), *rrm2b^+/−^* (*n* = 48) and *rrm2b^−/−^* (*n* = 14) larvae. Results are shown as mean ± SEM. (I) Swim bladder area (SBA) measurements of 5 dpf *rrm2b^+/+^* (*n* = 16), *rrm2b^+/−^* (*n* = 48) and *rrm2b^−/−^* (*n* = 14) larvae. Results are shown as mean ± SEM (statistical significance was determined with a Kruskal-Wallis test, ^*^^*^^*^^*^ = adj. *P* < 0.0001). (J) Representative images of 5 dpf *rrm2b^+/+^* larvae morphology (top panel) and 5 dpf *rrm2b^−/−^* larvae morphology (bottom panel). White arrow indicates uninflated swim bladder. (K) Relative mtDNA copy number of whole 3 dpf *rrm2b*^+/+^ & *rrm2b*^+/−^ (*n* = 13) and *rrm2b*^−/−^ (*n* = 14) larvae. Results are shown as mean ± SEM. (L) Relative mtDNA copy number in whole 5 dpf *rrm2b^+/+^* & *rrm2b^+/−^* larvae (*n* = 50) and *rrm2b^−/−^* larvae (*n* = 29). Results are shown as mean ± SEM. (M) Relative mtDNA copy number in the head and abdomen of 5 dpf *rrm2b^+/+^* & *rrm2b^+/−^* larvae (*n* = 21) and *rrm2b^−/−^* larvae (*n* = 16). Results are shown as mean ± SEM. (N) Relative mtDNA copy number in tail muscle of 5 dpf *rrm2b^+/+^* & *rrm2b^+/−^* larvae (*n* = 15) and *rrm2b^−/−^* larvae (*n* = 12) results are shown as mean ± SEM (statistical significance was determined with a Student’s t-test, ^*^ = *P* < 0.05). (O) Survival of *rrm2b* larvae beyond 5 dpf by genotype. 5 dpf: *rrm2b^+/+^*: 25% (*n* = 23), *rrm2b^+/−^*: 52% (*n* = 48), *rrm2b^−/−^*: 23% (*n* = 21). 7 dpf: *rrm2b^+/+^*: 18% (*n* = 6), *rrm2b^+/−^*: 58% (*n* = 19), *rrm2b^−/−^*: 24% (*n* = 8). 10 dpf: *rrm2b^+/+^*: 37% (*n* = 15), *rrm2b^+/−^*: 53% (*n* = 21), *rrm2b^−/−^*: 10% (*n* = 4). 12 dpf: *rrm2b^+/+^*: 42% (*n* = 15), *rrm2b^+/−^*: 50% (*n* = 18), *rrm2b^−/−^*: 8% (*n* = 3). 14 dpf: *rrm2b^+/+^*: 16% (*n* = 5), *rrm2b^+/−^*: 87% (*n* = 27), *rrm2b^−/−^*: 0% (*n* = 0). 17 dpf: *rrm2b^+/+^*: 36% (*n* = 9), *rrm2b^+/−^*: 60% (*n* = 15), *rrm2b^−/−^*: 4% (*n* = 1). 19 dpf: *rrm2b^+/+^*: 37% (*n* = 12), *rrm2b^+/−^*: 63% (*n* = 20), *rrm2b^−/−^*: 0% (*n* = 0). 21 dpf: *rrm2b^+/+^*: 60% (*n* = 21), *rrm2b^+/−^*: 40% (*n* = 14), *rrm2b^−/−^*: 0% (*n* = 0).

Initial characterisation of larvae up to 5 dpf was performed. First, a tail coiling assay showed no difference between *rrm2b* genotypes at 24 hpf, showing that early development up to 24 hpf is unaffected (Supplementary Fig. 2A). At 2 dpf, a touch evoked escape response assay showed that both mean escape velocity and total displacement are significantly reduced in *rrm2b^−/−^* larvae compared to *rrm2b^+/+^* and *rrm2b^+/−^* clutch mates, with reductions of up to 55% and 46% in swimming velocity and total distance moved respectively (Fig. 2A and B). Peak acceleration was unaffected (Fig. 2C), indicating that the force generating capacity of the *rrm2b^−/−^* larvae is unaffected, but the ability to sustain the escape response was altered, suggesting potential muscle fatigability.

At 5 dpf, five morphological parameters were quantified, standard length (SL), snout-vent length (SVL), head-tail angle (HTA), eye area (EA) and swim bladder area (SBA) as illustrated in Fig. 2D. We saw no difference in any of these parameters at 5 dpf between *rrm2b* genotypes (Fig. 2E–H). The swim bladder (SBA) of *rrm2b^−/−^* larvae was significantly smaller than both *rrm2b^+/+^* and *rrm2b^+/−^* clutchmates, with the majority being less than 0.05mm^2^ or ‘uninflated’ (Fig. 2I). Figure 2J illustrates the inflated (*rrm2b^+/+^)* vs uninflated (*rrm2b^−/−^*) swim bladder phenotype. To inflate the swim bladder, larvae perform a characteristic ‘swim up behaviour’, which requires coordination of the nervous system and muscle [31], any defects in these tissues may alter this activity.

### Analysing mtDNA copy number in *rrm2b^−/−^* zebrafish

To determine if mtDNA copy number, the main biomarker of mtDNA depletion syndromes, is altered in *rrm2b^−/−^* larvae, different time points and tissues were assessed for mtDNA content. Initially, we assessed whole larvae mtDNA copy number, and saw no significant difference between *rrm2b* genotypes at 3 and 5 dpf (Fig. 2K and L). As mtDNA depletion syndromes are tissue specific, with pathogenic *RRM2B* variants primarily affecting the CNS and skeletal muscle, we assessed mtDNA copy number in different tissues of 5 dpf *rrm2b* larvae. Heads and abdomen, containing the central nervous system and abdominal organs, were dissected from tails, which are primarily skeletal muscle, and mtDNA copy number was quantified. There was no difference in head/abdomen mtDNA copy number between *rrm2b* genotypes (Fig. 2M), but we saw a significantly lower mtDNA copy number in the tail muscle of 5 dpf *rrm2b^−/−^* larvae compared to control clutchmates. (Fig. 2N), indicating a tissue specific depletion of mtDNA in skeletal muscle.

To investigate mtDNA replication further in this model, ethidium bromide (EtBr) was used to artificially deplete mtDNA in *rrm2b* larvae between 0 dpf and 3 dpf before washing out and allowing mtDNA to repopulate up to 5 dpf. We saw no significant difference in whole larvae mtDNA copy number between EtBr treated genotypes after mtDNA depletion (Supplementary Fig. 2B).

Quantification of *rrm2b* genotypes up to 21 dpf shows *rrm2b^−/—^*larvae do not survive beyond 17 dpf, with a large loss between 7 dpf and 10 dpf (Fig. 2O). Both wild type and heterozygous fish (*rrm2b^+/+^* and *rrm2b^+/−^*) have no impairment in survival and are present in their approximate expected mendelian ratios.

Together, we show that *rrm2b^−/−^* larvae up to 5 dpf display characteristics of MDDS, with mild, but significant depletion of mtDNA in tail muscle, impaired escape response, uninflated swim bladders and a severely shortened lifespan.

### Deoxynucleoside supplementation alters mtDNA copy number in zebrafish larvae

**Figure 3:**
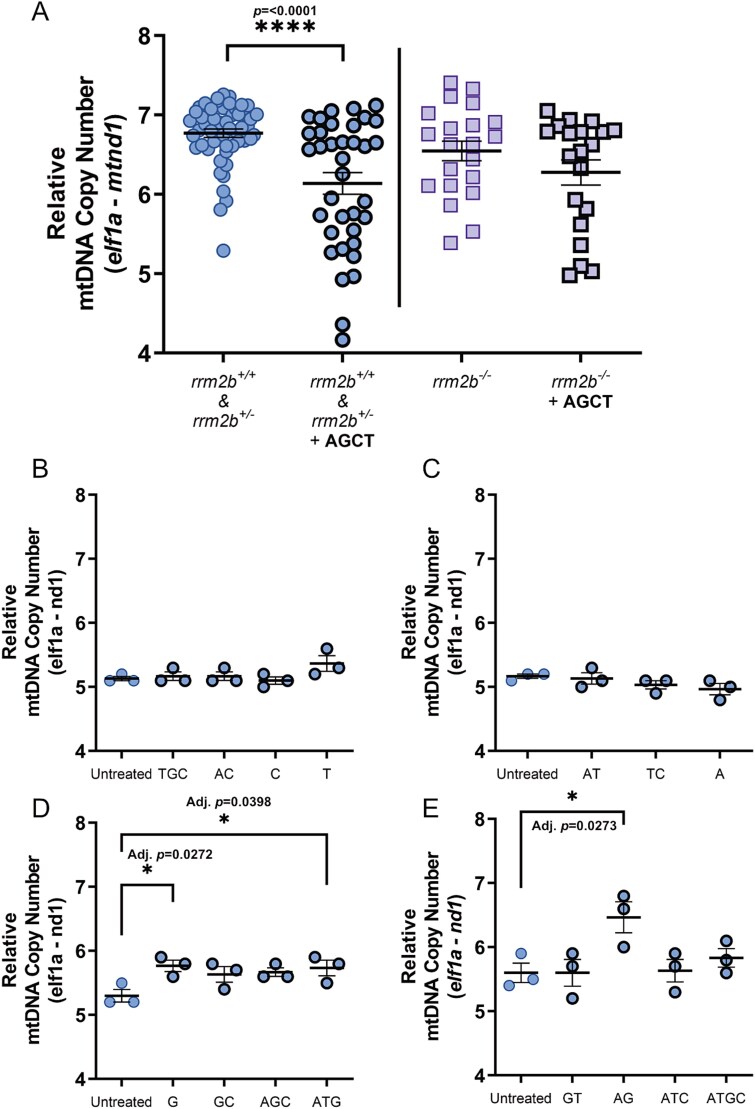
Deoxynucleoside supplementation in 5 dpf *rrm2b* and wild type larvae. A. Relative mtDNA copy number in 5 dpf *rrm2b* larvae treated between 0 dpf and 5 dpf with 50 μM of deoxynucleosides: dAdo (A), dGuo (G), dCtd (C) & dTmd (T) with 5 μM adenosine deaminase inhibitor, EHNA. Untreated 5 dpf *rrm2b^+/+^* & *rrm2b^+/−^* tail muscle (*n* = 54) (circles with thin border), treated 5 dpf *rrm2b^+/+^* & *rrm2b^+/−^* tail muscle (*n* = 36) (circles with thick border). Untreated 5 dpf *rrm2b^−/−^* tail muscle (*n* = 21) (squares with thin border), treated 5 dpf *rrm2b^−/−^* tail muscle (*n* = 20) (squares with thick border). Results are shown as mean ± SEM (statistical significance was determined with a Mann–Whitney U test, ^*^^*^^*^^*^P < 0.0001). B–E. Relative mtDNA copy number of pools of 10, 5 dpf WT whole larvae treated with 15 combinations of deoxynucleosides (circles with thick border) or untreated (circles with thin border). 5 μM of ENHA (deaminase inhibitor) was also supplemented with each deoxynucleoside combination. 3 pools of 10 larvae per treatment condition. Results are shown as mean ± SEM (statistical significance was determined with a one-way ANOVA, ^*^ = adj. *P* < 0.05).

To investigate the effect of deoxynucleoside supplementation on this model, we first treated *rrm2b* larvae with equimolar amounts of four deoxynucleosides (50 μM of 2′-Deoxyadenosine monohydrate (dAdo), 2’-Deoxyguanosine monohydrate (dGuo), 2′-Deoxycytidine (dCtd) & thymidine (dTmd)) and 5 μM erythro-9-(2-hydroxy-3-nonly) adenine (EHNA) hydrochloride (an adenosine deaminase inhibitor) between 0 dpf and 5 dpf. Analysis of mtDNA copy number in tail muscle after treatment showed a significantly lower mtDNA copy number in control *rrm2b^+/+^* and *rrm2b^+/−^* 5 dpf larvae, relative to untreated larvae (Fig. 3A), while treated mutant *rrm2b^−/−^* larvae have a mild, albeit not significant decrease in mtDNA copy number compared to untreated (Fig. 3A). This result suggests that deoxynucleoside supplementation in developing zebrafish larvae can potentially imbalance nucleotide pools with negative consequences on mtDNA levels. We thought a better understanding on how different combinations of deoxynucleosides affect mtDNA copy number in WT larvae would help inform the best treatment strategy.

Following on, we assessed the effect of 15 different deoxynucleoside combinations on developing wild type (AB strain) zebrafish mtDNA copy number. Between 0 dpf and 5 dpf, we observed three deoxynucleoside combinations, dGuo alone, dGuo & dAdo and dGuo, dAdo & dTmd significantly raise mtDNA copy number in pools (*n* = 10) of 5 dpf WT larvae, compared to untreated WT larvae (Fig. 3B-E). These results informed us of the combinations that may be most effective, or least toxic, to trial on the *rrm2b* larvae.

### Purine deoxynucleosides improve mtDNA copy number and movement in *rrm2b^−/−^* larvae

**Figure 4:**
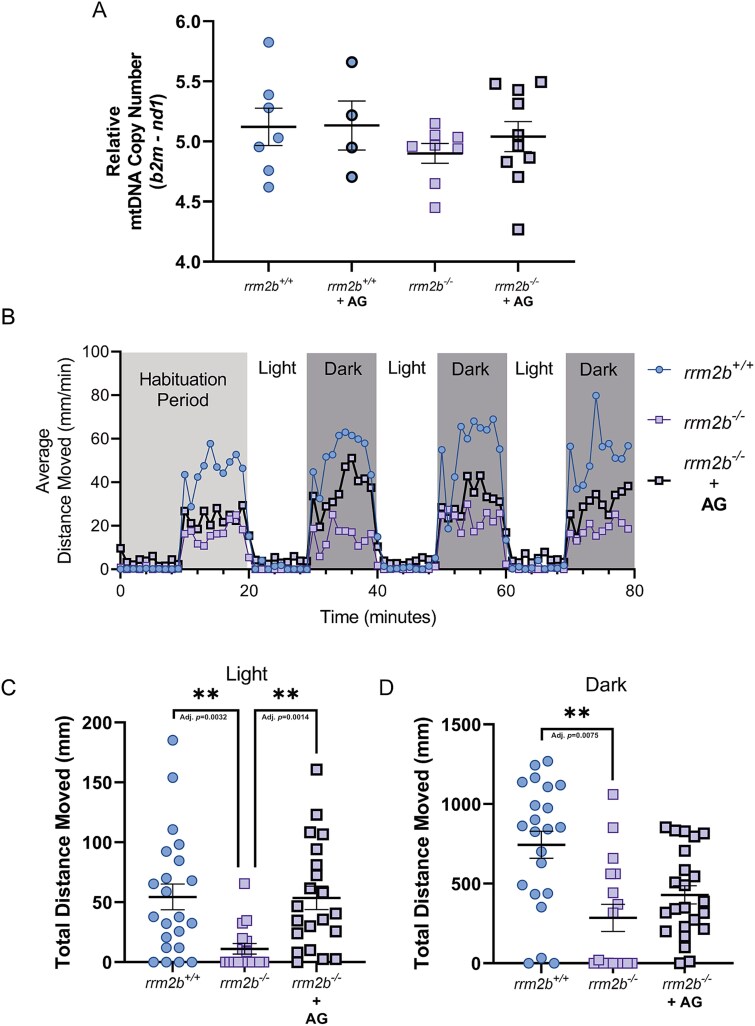
Assessment of purine deoxynucleoside supplementation in 5 dpf *rrm2b* larvae—.(A) Relative mtDNA copy number in whole 5 dpf *rrm2b* larvae, treated with either vehicle (DMSO) (*rrm2b^+/+^ n* = 7 (circles with thin border), *rrm2b^−/−^ n* = 9, (squares with thin border)) or purine deoxynucleosides (50 μM dAdo & dGuo + 5 μM ENHA in 0.5%DMSO) (*rrm2b^+/+^ n* = 4 (circles with thick border), *rrm2b^−/−^ n* = 10 (squares with thick border)) between 0 dpf and 5 dpf. Results are shown as mean ± SEM. (B) Average movement profile of 5 dpf vehicle treated *rrm2b^+/+^* (0.5% DMSO vehicle)(*n* = 22 (circles with thin border)), vehicle treated *rrm2b^−/−^* (0.5% DMSO vehicle)(*n* = 17 (squares with thin border)) and deoxynucleoside treated *rrm2b^−/−^* (50 μM dAdo & dGuo + 5 μM ENHA in 0.5%DMSO)(*n* = 21 (squares with thick border)) larvae during a light dark transition test (LDT). (C) Total distance moved in light periods of vehicle treated 5 dpf *rrm2b^+/+^* larvae (*n* = 22 (circles with thin border)), vehicle treated *rrm2b^−/−^* (0.5% DMSO vehicle)(*n* = 17 (squares with thin border)) and deoxynucleoside treated *rrm2b^−/−^* (50 μM dAdo & dGuo + 5 μM ENHA in 0.5%DMSO)(*n* = 21 (squares with thick border)) larvae during an LDT test. (I) Total distance moved in dark periods of vehicle treated 5 dpf *rrm2b^+/+^* larvae (*n* = 22 (circles with thin border), vehicle treated *rrm2b^−/−^* (0.5% DMSO vehicle)(*n* = 17 (squares with thin border) and deoxynucleoside treated *rrm2b^−/−^* (50 μM dAdo & dGuo + 5 μM ENHA in 0.5%DMSO)(*n* = 21 (squares with thick border)) larvae during an LDT test. Results are shown as mean ± SEM (statistical significance was determined with a one-way ANOVA, ^*^^*^P < 0.01).

Based on the identification that purine deoxynucleosides elevated mtDNA copy number in WT larval zebrafish, we supplemented both mutant and wild type *rrm2b* larvae with dAdo & dGuo between 0 dpf and 5 dpf. This was also the combination used previously to successfully treat a zebrafish model of *DGUOK*-related MDDS [29]. Supplementation of purine deoxynucleosides (50 μM dAdo & dGuo + 5 μM EHNA in 0.5% DMSO) to whole larvae between 0 dpf and 5 dpf showed no change in mtDNA copy number in *rrm2b^+/+^* larvae relative to untreated (0.5% DMSO) (Fig. 4A), avoiding the decrease we saw when supplemented with all four deoxynucleosides observed previously. We saw a mild elevation of mtDNA copy number in mutant *rrm2b^−/−^* larvae relative to vehicle treated larvae (Fig. 4A). However, due to the high mtDNA variability between larvae and low level of depletion in whole 5 dpf *rrm2b^−/−^* larvae, relative to *rrm2b^+/+^*, it is difficult to make any conclusions about how deoxynucleoside supplementation may affect mtDNA copy number in this model at 5 dpf.

To further study muscle function and deoxynucleoside supplementation in our fish model, a light/dark transition (LDT) assay was performed to establish if movement is affected in older larvae (5 dpf). The LDT assay involves alternating periods of 10 minutes of light or darkness, during which the distance moved of each larva is recorded. Typically, 5 dpf zebrafish larvae have increased movement in darkness and reduced movement in light. We observed, that relative to the 5 dpf *rrm2b^+/+^* larvae, 5 dpf *rrm2b^−/−^* mutant larvae have a noticeable reduction in the average distance moved per minute profile (Fig. 4B). Quantification of total distance moved in both light and dark confirms that 5 dpf *rrm2b^−/−^* larvae have significantly reduced movement compared to *rrm2b*^+/+^ larvae, in both the light and dark phases of the LDT assay (Fig. 4C and D). After treatment with purine deoxynucleosides (50 μM dAdo & dGuo + 5 μM EHNA in 0.5% DMSO), 5 dpf wild type *rrm2b^+/+^* fish have no significant change in their movement profiles or total distances moved (Supplementary Fig. 3A–C). There was however, a noticeable improvement in the movement profile of the *rrm2b^−/−^* larvae treated with deoxynucleosides (Fig. 4B) and when quantified, we saw a significant rescue of total distance moved in the light phase relative to untreated *rrm2b^−/−^* larvae (Fig. 4C), and an increase in total distance moved in *rrm2b^−/−^* larvae in the dark phase (a 42% decrease in movement vs a 62% decrease in the untreated *rrm2b^−/−^*). There was no significant difference between wild type untreated and *rrm2b^−/−^* treated fish (Fig. 4D). These results together demonstrate that deoxynucleoside supplementation can rescue mtDNA copy number and movement defect in *rrm2b^−/−^* larvae.

### Purine deoxynucleoside supplementation mitigates mtDNA depletion and lowers elevated lactate by altering nucleotide pools in 7 dpf *rrm2b^−/−^* larvae

**Figure 5:**
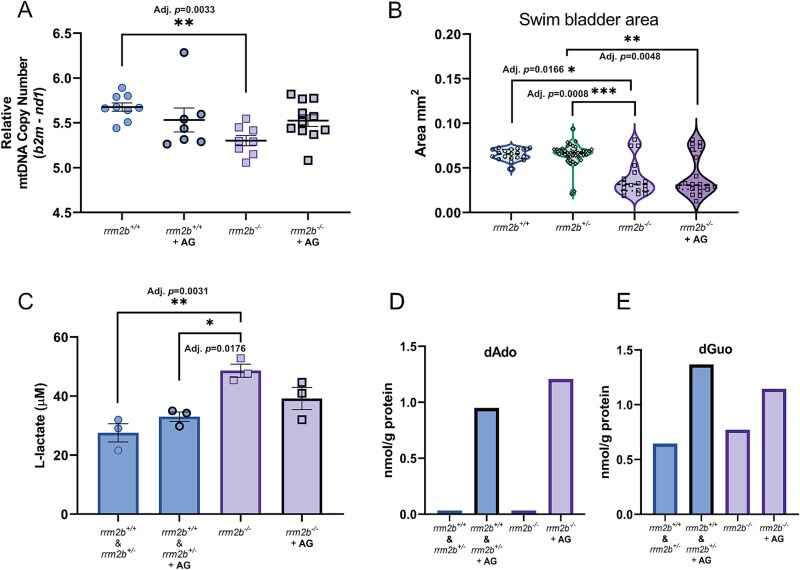
Assessment of purine deoxynucleoside supplementation in 7 dpf *rrm2b* larvae—. (A) Relative mtDNA copy number in vehicle treated (0.5% DMSO) 7dpf *rrm2b^+/+^* (*n* = 9)(circles with thin border) & *rrm2b^−/−^* (*n* = 8)(squares with thin border) larvae and purine deoxynucleoside treated (50 μM dAdo & dGuo +5 μM ENHA in 0.5%DMSO) 7 dpf *rrm2b^+/+^* (*n* = 7)(circles with thick border) & *rrm2b^−/−^* (*n* = 12)(squares with thick border) larvae. All results shown as mean ± SEM (statistical significance was determined with a Kruskal-Wallis test, ^*^^*^adj. *P* < 0.01). (B) Swim bladder area of 7 dpf vehicle treated (0.5% DMSO) *rrm2b^+/+^* (*n* = 19) (circles with thin border), *rrm2b^+/−^* (*n* = 40) (diamonds), *rrm2b^−/−^* (*n* = 18) (squares with thin border) and purine deoxynucleoside treated (50 μM dAdo & dGuo +5 μM ENHA in 0.5%DMSO) *rrm2b^−/−^* larvae (*n* = 20) (squares with thick border). All results are shown as mean ± SEM (statistical significance was determined with a Kruskal-Wallis test, ^*^ = adj. *P* < 0.05, ^*^^*^ = adj. *P* < 0.01, ^*^^*^^*^ = adj. p < 0.001). (C) L-lactate in 7 dpf vehicle treated (0.5% DMSO) *rrm2b^+/+^* & *rrm2b^+/−^* larvae (circles with thin border) and *rrm2b^−/−^* larvae (squares with thin border) and L-lactate in 7 dpf purine deoxynucleoside treated (50 μM dAdo & dGuo +5 μM ENHA in 0.5%DMSO) *rrm2b^+/+^* & *rrm2b^+/−^* larvae (circles with thick border) and *rrm2b^−/−^* larvae (squares with thick border). For all conditions n = 3 pools of 20, 7 dpf larvae. All results are shown as mean ± SEM (statistical significance was determined with a one-way ANOVA, ^*^ = adj. *P* < 0.05,^*^^*^ = adj. *P* < 0.01). (D) Deoxyadenosine concentration (nmol/g protein) in pools (*n* = 80) of 7 dpf vehicle treated (0.5% DMSO) or purine deoxynucleoside treated (50 μM dAdo & dGuo +5 μM ENHA in 0.5%DMSO) *rrm2b^+/+^* & *rrm2b^+/−^* and *rrm2b^−/−^* larvae. E. Deoxyguanosine concentration (nmol/g protein) in pools (*n* = 80) of 7 dpf vehicle treated (0.5% DMSO) or purine deoxynucleoside treated (50 μM dAdo & dGuo +5 μM ENHA in 0.5%DMSO) *rrm2b^+/+^* & *rrm2b^+/−^* and *rrm2b^−/−^* larvae. Deoxycytidine monophosphate, dTMP: Deoxythymidine monophosphate, TK2: Thymidine kinase 2.

Due to the large variation in mtDNA levels and low, tissue specific mtDNA depletion in 5 dpf *rrm2b^−/−^* larvae, we studied 7 dpf larvae to establish if increased age influences mtDNA depletion and other disease phenotypes. We first saw a significant depletion in mtDNA copy number in whole mutant *rrm2b^−/−^* larvae compared to *rrm2b^+/+^* larvae (Fig. 5A), greater than observed in 5 dpf *rrm2b^−/−^* tail muscle (Fig. 2N). Next, after treatment with purine deoxynucleosides up to 7 dpf (50 μM dAdo & dGuo + 5 μM EHNA in 0.5% DMSO), we saw a mitigation of mtDNA depletion in mutant *rrm2b^−/—^*larvae, where mtDNA copy number is not significantly different to untreated *rrm2b^+/+^* larvae (Fig. 5A). This result demonstrates that purine deoxynucleoside supplementation is able to prevent mtDNA depletion in this *RRM2B* MDDS zebrafish model.

Morphological parameters of 7 dpf larvae are similar to 5 dpf *rrm2b^−/−^* larvae, they are also lacking an inflated swim bladder (Fig. 5B & Supplementary Fig. 4A–D). Representative images of 7 dpf *rrm2b^−/−^* larvae demonstrate abnormal morphology (Supplementary Fig 4E and F). After treatment with deoxynucleosides up to 7 dpf (50 μM dAdo & dGuo + 5 μM EHNA in 0.5% DMSO), we see no effect on any of the morphological parameters (SL, SVL or HTA, or EA) in 7 dpf *rrm2b^−/−^* larvae after deoxynucleoside treatment (Supplementary Fig. 4A–D). The proportion of inflated swim bladders (SB) (SBA > 0.05 mm^2^) in the mutant *rrm2b^−/−^* larvae after deoxynucleoside treatment increased (30% inflated) compared to vehicle treated 7 dpf *rrm2b^+/+^* larvae (16% inflated) (Fig. 5B), potentially indicating a treatment effect.

As lactic acidosis is often seen in patients with *RRM2B-*related disease, and in many other mitochondrial diseases, we looked to establish if lactate levels change in 7 dpf *rrm2b* larvae. Analysis of L-lactate in pools (*n* = 30) of 7 dpf *rrm2b* larvae showed a significant elevation in vehicle treated mutant 7 dpf *rrm2b^−/−^* larvae relative to vehicle treated *rrm2b^+/+^* & *rrm2b^+/−^* larvae of 76% (Fig. 5C). Subsequently, we observe that after treatment with purine deoxynucleosides (50 μM dAdo & dGuo +5 μM EHNA in 0.5%DMSO) for 7 days the L-lactate level of 7 dpf *rrm2b*^−/−^ larvae is reduced, dropping by 34%, not significantly elevated compared to control larvae (Fig. 5C), adding again to the evidence that deoxynucleoside supplementation is able to attenuate disease associated phenotypes in this model.

Finally, to confirm that the supplemented nucleotides are entering the larvae and contributing to the phenotype attenuation, we quantified deoxynucleosides in pools (*n* = 80) of deoxynucleoside treated and untreated whole 7 dpf *rrm2b* larvae via LC-MRM/MS. We confirmed that both dAdo and dGuo are elevated in both the mutant and control deoxynucleoside treated *rrm2b* larvae, indicating the deoxynucleosides pass into larvae (Fig. 5D and E). Together, these results suggest that by increasing purine deoxynucleosides in *rrm2b^−/−^* larvae up to 7 dpf, it is possible to modify their disease associated phenotypes, supporting deoxynucleoside supplementation as a potential therapeutic option for patients with MDDS.

## Discussion

Mitochondrial DNA depletion syndromes are severe and life-threatening diseases without an approved effective treatment. Progress has been made recently where pyrimidine deoxynucleoside supplementation has rescued mtDNA depletion and stalled or reversed disease progression in patients with pathogenic *TK2* variants [21, 32]. However, not much data are available on the efficacy of deoxynucleoside therapies for other forms of MDDS. To advance treatments for MDDS, here we have developed a novel zebrafish model of *RRM2B-*related disease, where larvae carrying a homozygous nonsense mutation in *rrm2b* present with impaired movement, reduced mtDNA copy number and elevated lactate, all of which are mitigated with deoxynucleoside supplementation.

Mutant larvae (*rrm2b^−/−^*) at 2 dpf have a significantly attenuated touch evoked escape response, demonstrating that while mutant larvae are able to reach peak acceleration, they struggle to sustain movement beyond this point. This difference suggests that the force generating capacity of the muscle is normal but the ability to sustain movement beyond this is impaired due to potential fatigability. The movement impairment is further built on at 5 dpf, where we observe that light stimulated prolonged movement is also significantly affected in mutants, mirroring early onset muscle weakness seen in patients. Similarly reduced movement has also been observed in other models of MDDS including a *Tk2* and *Polg* mutant mouse models [33, 34]. Further, at 5 dpf, it’s apparent that a large proportion of mutant *rrm2b^−/−^* larvae lack inflated swim bladders. Normally, before 5 dpf, larvae perform ‘swim-up behaviour’ and take in air at the water surface to inflate their posterior swim bladder which involves coordination of muscle and nervous system [31]. Defects in the ability of larvae to perform this behaviour have previously been attributed to defects in mitochondrial function, including zebrafish with mutations in *mpv17*, another MDDS disease gene [35, 36]. To further develop the relevance of the model, we subsequently showed that 7 dpf *rrm2b^−/−^* larvae have elevated L-lactate, adding to the clinical relevance of the model. We can assume this combination of disease associated abnormalities leads to a shorter lifespan of *rrm2b^−/−^* larvae, where the majority die by 14 dpf. Together, this gives us an indication that the homozygous *rrm2b* mutation has a detrimental effect on zebrafish larvae with many characteristics matching *RRM2B* defect in patients. These phenotypes allowed us to study the efficacy of deoxynucleoside supplementation on an *in vivo* model of *RRM2B* MDDS.

As MDDS manifest in a tissue specific manner, we first investigated mtDNA copy number in two different anatomical areas, such as the tail (primarily skeletal muscle) and the abdomen and head which contain variety of tissues. Here we see a mild, but significant depletion of mtDNA in the tail muscle of homozygous larvae, while no difference has been detected in the head & tail, implying a tissue specific depletion of mtDNA in skeletal muscle, also observed in *Rrm2b* KO mice and patients [11, 18]. While this depletion of mtDNA in tail muscle at 5 dpf is important, it is a relatively small difference, and due to the variation in mtDNA copy number at this stage it would have been difficult to observe any meaningful effect of treatment. We think that this is due to the fact, that the fast development of zebrafish between 0 and 5 dpf involves rapid cell division and in dividing cells mitochondrial nucleotide pools are replenished from the cytosol. Nucleotides are synthesise in large amounts in the cytosol by RNR, using *Rrm2* as the small RNR complex sub-unit, making *rrm2b* semi-redundant at this stage, especially when analysing a mixture of tissues. As development slows, post-mitotic tissues become more reliant on *rrm2b*, and in support of this hypothesis, at 7 dpf we detected a greater depletion of mtDNA in whole mutant larvae. This greater depletion at 7 dpf allowed us to better determine the efficacy of deoxynucleoside supplementation at modulating mtDNA copy number in *rrm2b* larvae.

Initially we treated larvae up to 5 dpf with an equimolar combination of all four deoxynucleosides (dAdo, dGuo, dCtd & dTmd), as RNR is implicated in the regulation of all four deoxynucleosides, however we were surprised to see a reduction of mtDNA in tail muscle of both wild type and heterozygous larvae after treatment. Nucleotide pools are finely balanced and instances of elevated mitochondrial dTTP have been shown to cause reduced mtDNA copy number in patients with MNGIE, due to pathogenic variants in *TYMP* [37] and high equimolar concentrations of supplemented deoxynucleosides have been shown to cause toxicity *in vitro* [27]. Frisk *et al*. measured the nucleotide pools of zebrafish larvae between fertilisation and 6 dpf and saw that over the course of development the level of purine nucleotide levels decrease faster compared to pyrimidine nucleotides [38], suggesting supplementing excess pyrimidine deoxynucleosides throughout the first few days of development may lead to imbalances in nucleotide pools, which could explain the reduction of mtDNA we observed. The subsequent pilot study in pools of WT larvae with different combinations of deoxynucleosides identified purine deoxynucleosides are the most effective at elevating mtDNA copy number in larvae up to 5 dpf. Additionally, this combination of deoxynucleosides was previously shown the be effective at modulating mtDNA copy number in another model of *MDDS* [29], therefore moving forward we performed treatment only with dAdo and dGuo. As mentioned, due to the small level of mtDNA depletion and high variability at 5 dpf, purine deoxynucleoside supplementation had no conclusive effect on the mtDNA copy number of 5 dpf *rrm2b^−/−^* larvae. Despite this, we saw a partial rescue of the movement defect, the first indication that deoxynucleoside supplementation can mitigate the disease associated symptoms in this model. This was expanded further at 7 dpf, where we see a prevention of mtDNA depletion and lowered lactate in treated *rrm2b* mutants, providing evidence that we can not only prevent mtDNA depletion with deoxynucleoside supplementation, but also demonstrate a functional benefit.

While we demonstrate that supplementation of dAdo and dGuo is able to mitigate the effects of *rrm2b* KO in developing zebrafish larvae, this may not directly translate to what may be effective in treating patients. Supplemented dAdo is catabolised rapidly by adenosine deaminase (ADA) [39, 40] and here we co-supplement with EHNA, an ADA inhibitor, to increase bioavailability of the supplemented dAdo. However, it has been shown *in vivo* that supplementation of deaminase inhibitors may be toxic, as when tetrahydrouridine (THU), an inhibitor of cytidine deaminase, was co-administered with dCMP + dTMP in a TK2 KO mouse model, it resulted in reduced survival compared to dCMP + dTMP alone [23]. Further, mutations in ADA are linked to immune system dysfunction due to toxic levels of dAdo [41] indicating that co-supplementing patients with deaminase inhibitors may not be viable and alternative approaches to increase substrate bioavailability have to be explored.

In addition, the best effective dose and combination of deoxynucleosides in patients may differ from zebrafish. This is illustrated by differences in nucleotide metabolism between species, and also supported by our data with different deoxynucleoside combinations in zebrafish. Therefore more human studies are needed to find out the real benefit of deoxynucleoside supplementation in patients with different gene defects leading to mitochondrial depletion.

In summary, previous studies into deoxynucleoside supplementation as a therapy for mitochondrial disease due to pathogenic *RRM2B* variants have been performed on *in vitro* models that focus mainly on manipulation of mtDNA copy number [28]. Here we build on this evidence for the first time in an *in vivo* model of *RRM2B* MDDS, also demonstrating an amelioration of functional defects including improved movement and lower lactate production. Together this data further adds to the growing evidence that deoxynucleoside supplementation is a valid therapeutic approach for treating patients with pathogenic *RRM2B* variants and possible also for other forms of mitochondrial diseases caused by mtDNA depletion.

## Materials and methods

For a full and detailed materials and methods section, please refer to Supplementary file 1.

### Generation of *rrm2b* mutant zebrafish

Between 0 dpf—5 dpf zebrafish were grown at 28*°C* in plastic petri dishes. Target sequence of *rrm2b* was identified using CRISPRscan (www.crisprscan.org) and were chosen based on their efficiency score, location on the gene (avoiding exon 1) and no or low off-target binding. All oligos were produced by Merck/Sigma Aldrich. Oligos used to generate sgRNA: *rrm2b* exon 3 41 sgRNA top strand oligo—taatacgactcactataGGGGATAGTCAATGAGAACCgttttagagctagaa, universal bottom strand ultramer (which anneals to the designed top strand oligo)—5′AAAAGCACCGACTCGGTGCCACTTTTTCAAGTTGATAACGGACTAGCCTTATTTTAACTTGCTATTTCTAGCTCTAAAAC-3′. F0 generation were outcrossed with WT (AB strain) at 3 months of age and a sample of individual embryos collected at 5 dpf and DNA extracted. Once heterozygous mutations of interest were identified, the remaining clutchmates were grown to adults and genotyped, those carrying the mutations of interest are the F1 generation. Following a subsequent outcross with WT (TL) strain the F2 generation was raised, genotyped and in-crossed to produce experimental larvae. Genotyping was performed on genomic DNA extracted from adult tail clips or whole larvae via PCR using oligos flanking the mutation site (oligo sequences in Table 1).

**Table 1:** Oligo sequences.

**Gene target**	**Forward Primer (5′-3′)**	**Reverse Primer (5′-3′)**	**Application**
*rrm2b-exon 3*	CGGCCTGAAGTCTGAAGAGA	CCTCCTGACTGAACCTCTGG	Genotyping
*elf1a*	AAGCCGCTGAGGTAAGCGTTCAAC	TTGAGCCGAGAAACGCGTGCTG	mtDNA copy number
*mt-nd1*	CCACTTAATTAACCCCCTAGCC	ATGTTTGTGGGGGTAGACCA	mtDNA copy number
*b2m*	CGCCTGAAAACTACGTTCTACAC	ACTTTCGGAGTGGCTGAAAA	mtDNA copy number

### Relative mtDNA copy number

Relative mtDNA copy number was measured using a protocol established previously [42]. Primers targeting the mitochondrial gene NADH dehydrogenase-1 (*mt-nd1*) and a nuclear gene, elongation factor 1 (*elf1a*) or beta-2-microglobulin (*b2m*) were used (Table 1). Briefly, DNA was extracted from individual or pools of larvae by incubating samples in lysis buffer (25 mM NaOH, 0.2 mM EDTA), for 30 minutes at 95°C before neutralising with 40 mM Tris–HCl solution. DNA was diluted to 2 ng/μl per sample and a qPCR assay performed. Each reaction consisted of: 12.5 μl Sso Advanced Universal SYBR Green Supermix (Bio-Rad), 1.25 μl forward primer (10 μM), 1.25 μM reverse primer (10 μM), 9 μl nuclease free water and 1 μl DNA (2 ng/μl). Reactions were performed in triplicate per sample for each target, nuclear and mitochondrial, using a Bio-Rad CFX96 under the following program: 95°C—3 minutes (initial denaturation), followed by 40 cycles of 95°C—10 seconds & 60°C—30 seconds. A subsequent melt curve analysis was performed to determine single target amplification. The relative mtDNA copy number was calculated as ΔCt (nuclear gene Ct—mitochondrial gene Ct).

### Immunoblotting

Equal amounts of protein (between 10—20 μg) was mixed with 7.5 μl NuPage LDS sample buffer (4X), 3 μl NuPAGE Sample Reducing Agent (10X) and the remaining volume to 30uL with deionised water. Primary antibodies used include anti-RRM2B (SAB1405069) and anti-alpha Tubulin (GTX124303),

### Deoxynucleoside supplementation

Deoxyribonucleosides: 2-deoxyadenosine monohydrate (Sigma, D7400), 2′-deoxyguanosine monohydrate (Sigma, D7145), 2-deoxycytidine (Sigma, D3897) & thymidine (Sigma, T9250) were dissolved in either E3 or DMSO to a stock concentration of 100 mM (200X) and frozen at −20°C. Adenosine deaminase inhibitor, EHNA hydrochloride (Sigma, E114), was dissolved in E3 or DMSO to a stock concentration of 10 mM (200X) and frozen at −20°C. Embryos were supplemented with deoxynucleosides at 50 μM (1X) and ENHA hydrochloride at 5 μM (1X) in a volume of 30 mL of E3 medium. If deoxynucleosides dissolved in DMSO were used, control dishes were supplemented with DMSO to 0.5% concentration. Quantification of deoxynucleosides was performed using LC-MRM/MS by UVic-Genome bc Proteomics Centre, Victoria, Canada.

### Light/dark transition test

At 4 dpf, larvae were placed into a 96 well transparent microtiter plate with 200 μl of E3 medium/treatment medium and kept overnight at 28°C to acclimatise to the plate. The following day, using a Zantiks MWP system (Zantiks Ltd), movement was recorded over an 80-minute period with alternating periods of 10 minutes light and 10 minutes dark. For quantification, only movement in the first 5 minutes of cycles 2-4 was used to allow for initial habituation to the stimuli and to avoids desensitisation to the stimuli.

### L-lactate measurement

To measure L-lactate in 7 dpf larvae, 3 pools of 20 larvae were collected per treatment condition and following the manufacturers colorimetric protocol, L-lactate measured immediately using the L-Lactate Assay Kit (Abcam, ab65330) with the Deproteinizing Sample Preparation Kit—TCA (ab204708).

### Statistical analysis

All statistical analysis was performed in Graph Pad Prism v8.4.3 (more details in Supplementary information). To ensure technical accuracy of assays, a minimum of three technical replicates were performed per biological replicate.

### Ethics declaration

All zebrafish experiments carried out were carried out in accordance with UK Home Office Guidelines, UK Animals (Scientific Procedures) Act 1986 and with approval of the local University of Cambridge ethics committee. Adult zebrafish (>5dpf) were maintained under project licence P1CG6E735.

## Supplementary Material

Supplementary_Data_ddaf047
