# Supplementary material for: Deoxynucleoside supplementation ameliorates the disease associated phenotypes in a zebrafish model of RRM2B mtDNA depletion syndrome
Source: Hum Mol Genet. 2025 Apr 11;34(11):967–77. doi: 10.1093/hmg/ddaf047 (PMC12085779; doi:10.1093/hmg/ddaf047)
Supplement: Supplementary_Data_ddaf047 [file supplementary_data_ddaf047.docx]

**Supplementary Data**

**Supplementary figure 1 -** **Multiple sequence alignment of the *RRM2B* gene from 5 different model species.** DANRE: *Danio rerio* (Zebrafish), XENLA: *Xenopus laevis* (African clawed frog), HUMAN: *Homo sapiens* (Human), MOUSE: *Mus musculus* (House mouse), RAT: *Rattus norvegicus* (Brown rat). (Key: * (asterisk): positions where a single residue is conserved, : (colon): positions where residues share strongly similar properties, .(full stop): positions where residues share weakly similar properties.


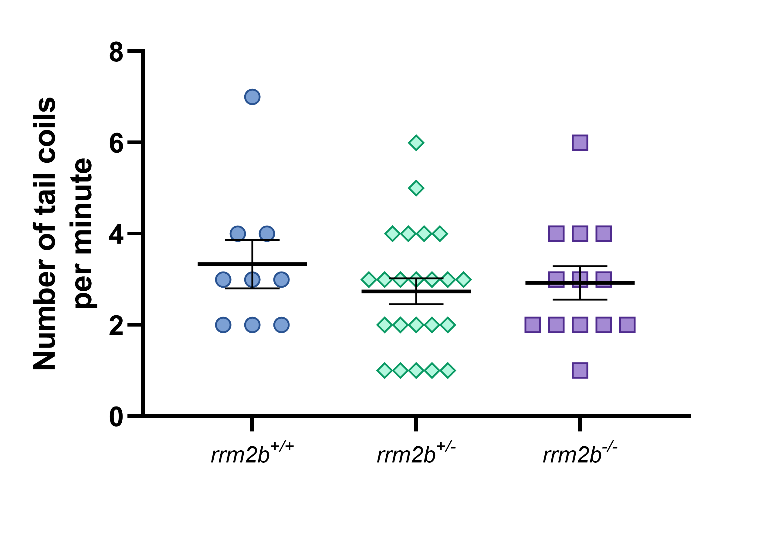


**A.**


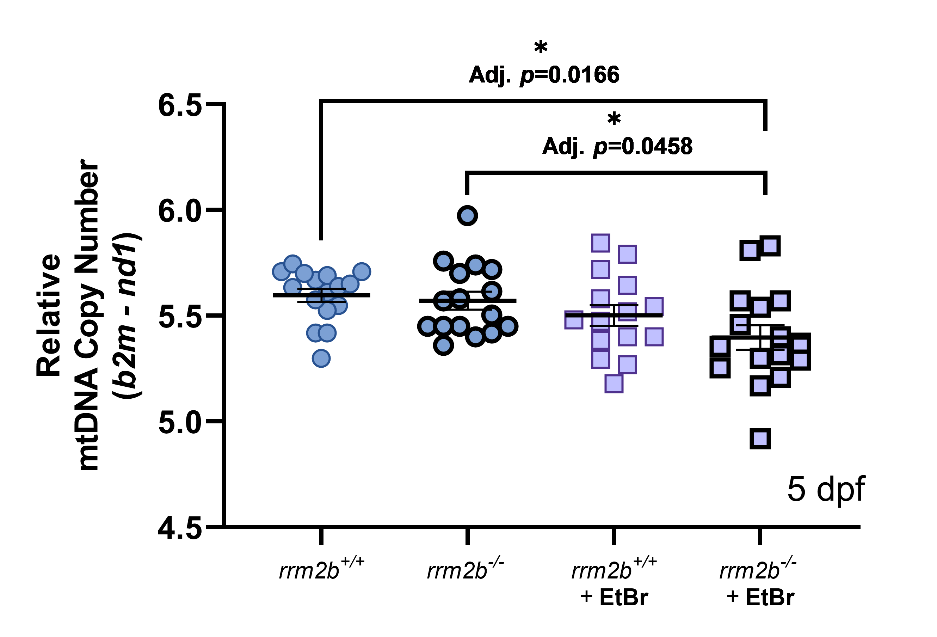


**B.**

**Supplementary Figure 2 –** **A**. **Tail coiling in 24 hpf, rrm2b larvae**. 24 hpf rrm2b^+/+^ (n=9), rrm2b^+/-^ (n=23) and rrm2b^-/-^ (n=13) larvae tail flicks over the period of 1 minute. **B. Relative mtDNA copy number between whole 5 dpf rrm2b larvae with or without EtBr treatment to 3 dpf.** Relative mtDNA copy number of untreated whole 5 dpf rrm2b^+/+^ larvae (n=16), untreated whole 5 dpf rrm2b^-/-^ larvae (n=16) and whole 5 dpf rrm2b^+/+^ (n=15) and rrm2b^-/-^ larvae (n=16) treated with EtBr until 3 dpf. Results are shown as mean ± SEM (Statistical significance was determined with a one-way ANOVA, *adj. p<0.05).

**
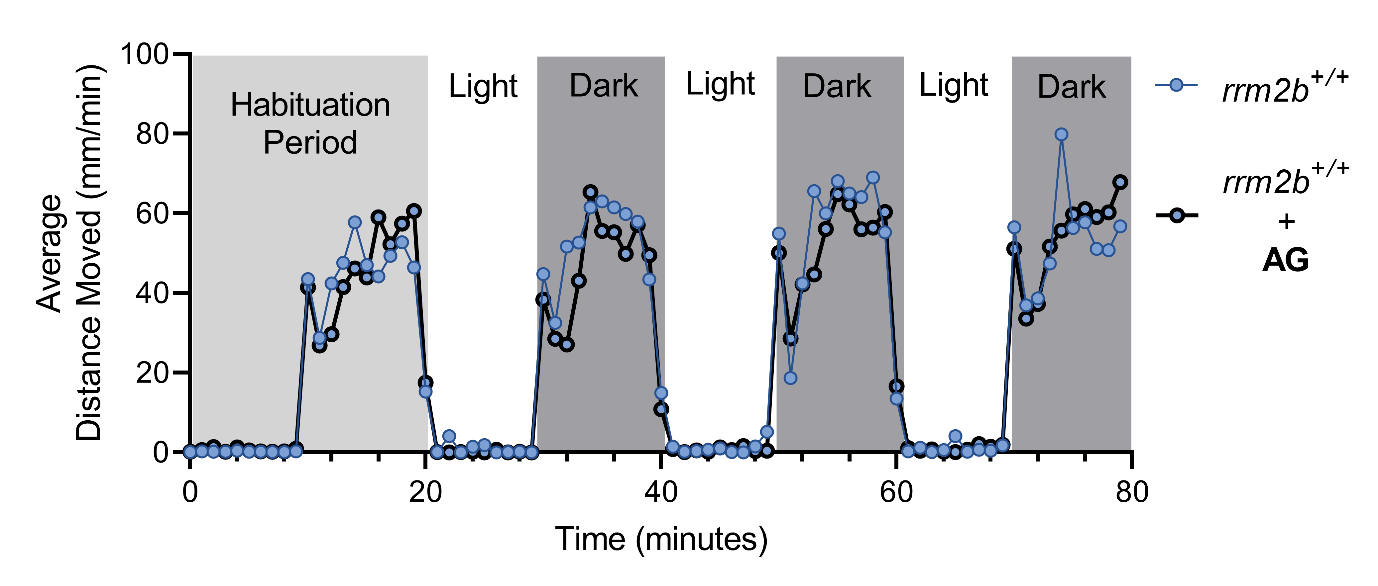
**

**A.**

**C.**

**B.**

**
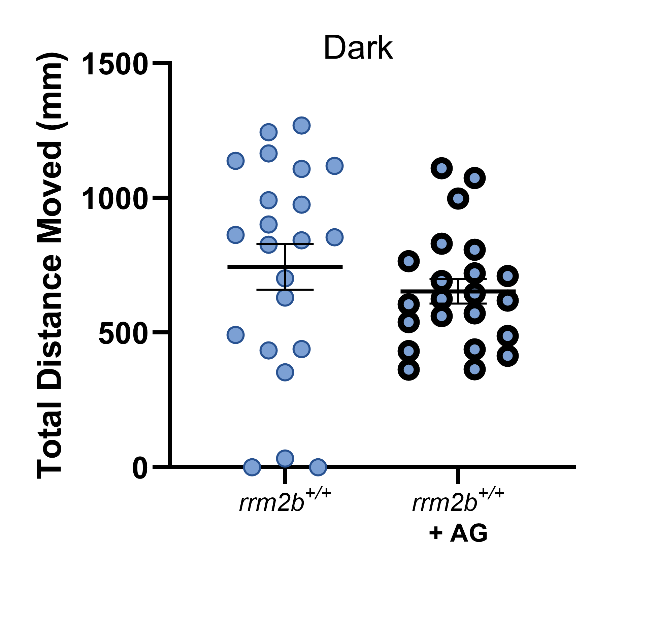

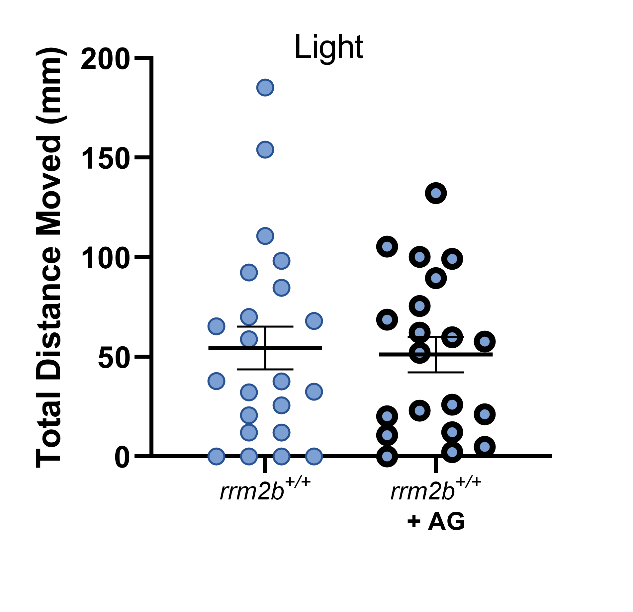
**

**Supplementary Figure 3 –** **A. Movement profiles and total distance moved of 5 dpf *rrm2b^+/+^* larvae from a LDT test after purine nucleoside treatment. A.** Average movement profile of 5 dpf *rrm2b^+/+^* larvae from a LDT test with or without nucleoside treatment. **B.** Total distance moved of 5 dpf *rrm2b^+/+^* larvae in light during an LDT test with (n=20) or without nucleoside treatment (n=22). **C.** Total distance moved of 5 dpf *rrm2b^+/+^* larvae in darkness during an LDT test with (n=20) or without nucleoside treatment (n=22). Results are shown as mean ± SEM.


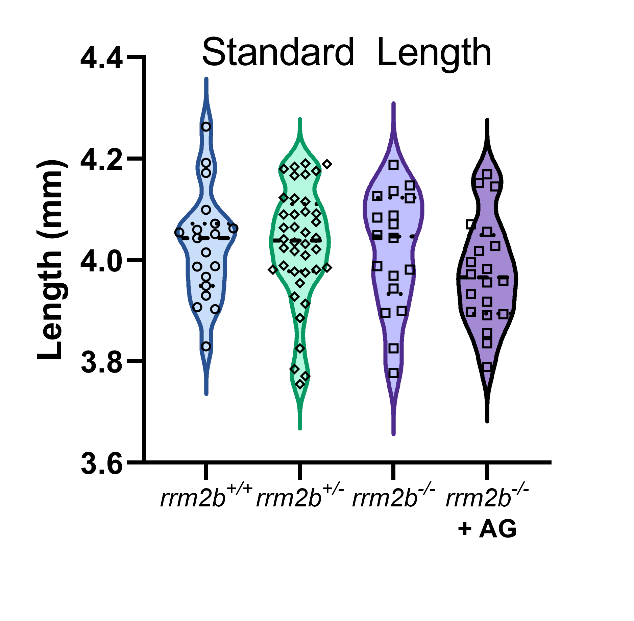


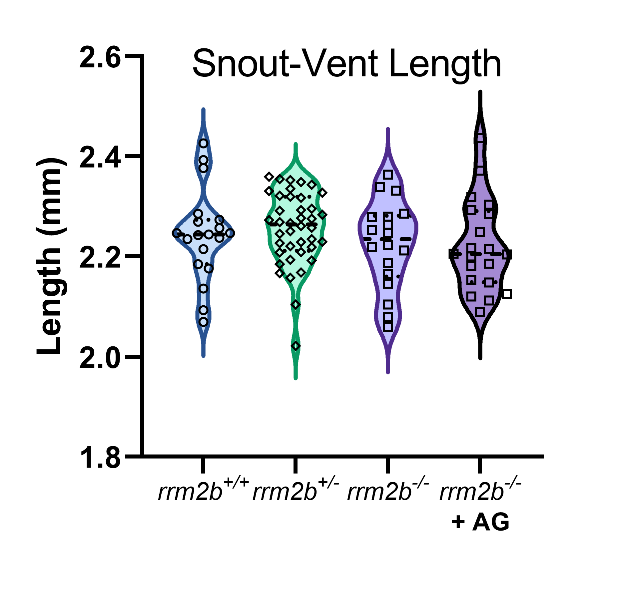


**B.**

**A..**


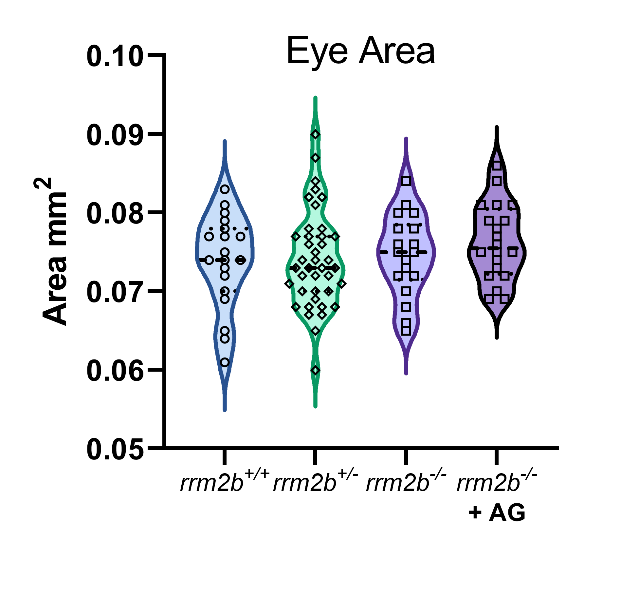


**D..**

**C..**


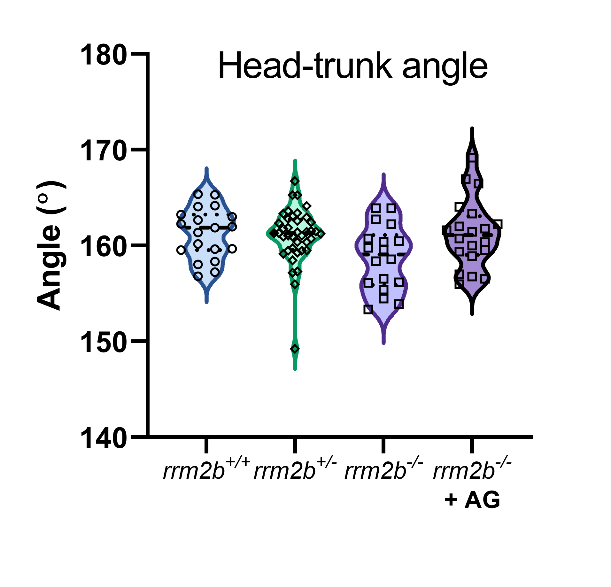


**E.**

**F.**


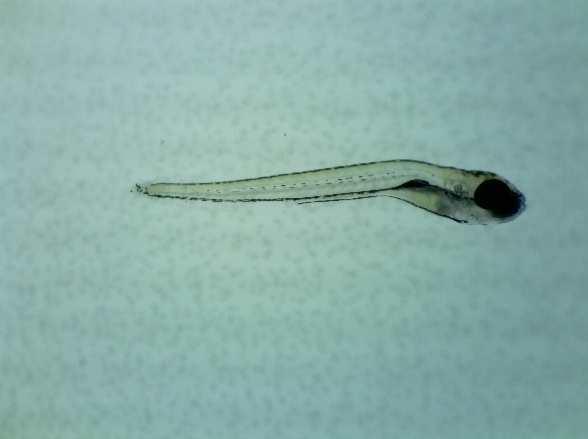


*rrm2b^-/-^*


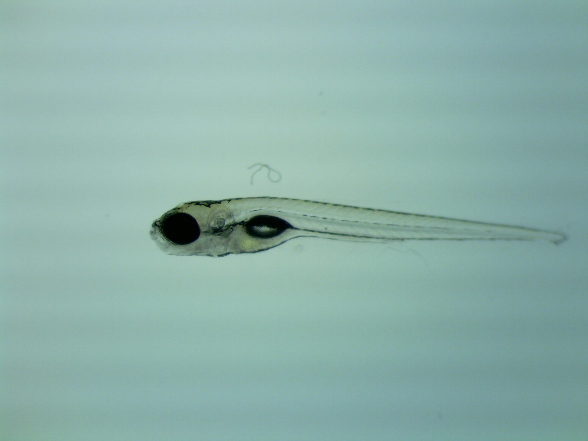


*rrm2b^+/+^*

**Supplementary figure 4 - Morphological analysis of 7 dpf untreated and nucleoside treated *rrm2b* larvae.** (**A**) Standard length of 7dpf *rrm2b* larvae (n=19/40/18/20). (**B**) Snout to vent length of 7 dpf *rrm2b* larvae (n=19/40/18/20). (**C**) Head – trunk angle of 7 dpf *rrm2b* larvae (n=19/40/18/20). (**D**) Eye area of 7 dpf *rrm2b* larvae (n=19/40/18/20). All results are shown as mean ± SEM. (**E**) Representative image of a 7 dpf *rrm2b^+/+^* larvae. (**F**) Representative image of a 7 dpf *rrm2b^-/-^* larvae.

**Supplementary Methods**

**Zebrafish Husbandry**

Between 0 dpf – 5 dpf zebrafish were grown at 28*°C* in plastic petri dishes at no more than 30 per dish in ~ 30mL of E3 medium, a standard medium used to grow embryonic and larval zebrafish. A 1X solution consists of: 5mM NaCl, 0.17mM KCl, 0.33mM CaCl2, 0.33mM MgSO4 in deionised water.

Larvae older than 5 dpf, juveniles and adults were grown in university aquatics facilities on a light cycle of 14 hrs light – 10 hrs dark. Larve between 5 dpf and 12 dpf were fed live paramecia with fish older fed a mix of brine shrimp and powdered formula.

**Breeding**

Breeding events were prepared the night prior to breeding, with group tanks being set-up at a 1:2 male to female ratio. Dividers were placed between the males and females and tanks placed on darkened bases overnight. The morning of the breeding, dividers were removed, and fish allowed to pair. Fertilised embryos were collected, and adults placed immediately back into their holding tanks. Collected embryos were checked for quality, and healthy embryos moved to petri dishes with E3 medium and incubated at 28*°C.*

**Sequence Alignment**

Multiple sequence alignments were performed using the ‘Clustal Omega’ sequence alignment tool from EMBL-EBI (https://www.ebi.ac.uk/Tools/msa/clustalo/) with ‘Clustal W with character counts’ as the output format.

**Single guide RNA (sgRNA) design and synthesis**

Target sequence was identified using CRISPRscan (Moreno-Mateos *et al.*, 2015) ([www.crisprscan.org](http://www.crisprscan.org)) and were chosen based on their efficiency score, location on the gene (avoiding exon 1) and no or low off-target binding. All oligos were produced by Merck/Sigma Aldrich. *rrm2b* exon 3 41 sgRNA top strand oligo – taatacgactcactataGGGGATAGTCAATGAGAACCgttttagagctagaa, universal bottom strand ultramer (which anneals to the designed top strand oligo) - 5′AAAAGCACCGACTCGGTGCCACTTTTTCAAGTTGATAACGGACTAGCCTTATTTTAACTTGCTATTTCTAGCTCTAAAAC-3′. To generate a template for *in vitro* transcription of sgRNA the following reaction was prepared for each sgRNA, a modified version from (Varshney *et al.*, 2016) (4 reactions per sgRNA). 5x MyTaq Buffer (Bioline) 5µL, top strand oligo (100µM) 2uL, universal bottom strand ultramer (100µM) 2µL, MyTaq DNA polymerase (Bioline) 0.2µL, nuclease free water (Ambion) 15.8µL. Templates were annealed in a thermocycler (Bio-Rad iCycler) under the following conditions: 95°C (5 minutes), 89°C, (15s), 83°C (15s), 77°C (15s), 71°C (15s), 65°C (15s), 59°C (15s), 53°C (15s), 50°C (10 minutes) and 72°C (10 minutes). Annealed products were pooled and purified using a Qiagen QIAquick PCR purification kit (Qiagen) according to manufacturer’s instructions. Purified sgRNA template was eluted in 30uL of nuclease free water (Ambion) and concentration determined with a nanodrop 2000 spectrophotometer (ThermoFisher). Samples were validated on a 4% agarose gel, with the expected product size around 120 base pairs. sgRNA was synthesised using the MEGAshort T7 kit (ThermoFisher), 2 reactions per sgRNA, using 8µL of purified sgRNA template and following the manufacturer’s instructions. After overnight incubation at 37°C products were treated with DNase (TURBO DNA-free kit (Ambion) and pooled before purification with the mirVana miRNA isolation kit with phenol (Ambion), from step F of the manufacturers protocol (final RNA isolation step), eluting in 50uL of nuclease free water. sgRNA size was validated on a 1% agarose gel and aliquoted and stored at -80°C until the morning of mutagenesis injections.

**Zebrafish Mutagenesis**

On the morning of mutagenesis injections ribonucleoproteins (RNP) were assembled by combining the following: 10x Cas9 Buffer (NEB) 1µL, Cas9 protein (2µM) with nuclear localisation signal (NLS) (NEB) 2µL, sgRNA (300ng/µL), 2M KCl 1.5µL, 0.5% phenol red (for droplet visualisation) 1µL and nuclease free water (Ambion) to 10µL total volume. RNP mix was incubated at 37°C for 10 minutes before keeping at 4°C until injections. Wild type (TL strain) zebrafish were paired in breeding tanks and embryos collected as soon as possible after spawning. Using a microinjector with borosilicate glass micropipettes, 0.5nL of assembled RNP injection mix was injected into newly fertilized 1-cell stage embryos as close to the yolk-cell boundary as possible. Following injections embryos were placed in petri dishes with E3 medium at no more than 30 per dish in a 27-28°C incubator until 5 dpf, when they are moved into the facility nursery tanks.

**Mutagenesis Validation**

F0 generation were outcrossed with WT (AB strain) at 3 months of age and a sample of individual embryos collected at 5 dpf and DNA extracted via alkaline lysis extraction (25mM NaOH, 0.2mM EDTA - lysis buffer, 40mM Tris-HCl - neutralisation buffer). Primers flanking the mutagenesis target site (oligo sequences in table 1) were used to amplify the target region via PCR, followed by sanger sequencing to identify any mutations of interest. Once heterozygous mutations of interest were identified, the remaining clutchmates were grown to adults and genotyped, those carrying the mutations of interest are the F1 generation. Following a subsequent outcross with WT (TL) strain the F2 generation was raised, genotyped and in-crossed to produce experimental larvae.

**Sanger sequencing**

Sequencing was outsourced to Eurofins Genomics.

**DNA sequence analysis**

DNA sequences were analysed using Chromas (version 2.6.6) and NCBI standard nucleotide basic local alignment search tool (BLAST) to identify mutations in the target region. For analysis of F1 heterozygous sequences the Poly Peak Parser web tool was used to separate out the different sequences and highlight mutations (Hill *et al.*, 2014) (http://yosttools.genetics.utah.edu/PolyPeakParser/). Mutations were confirmed manually, counting the bases, to ensure the mutation given was correct.

To determine the consequence of mutations, EMBOSS Transeq (https://www.ebi.ac.uk/Tools/st/emboss_transeq/) was used to predict the peptide sequences resulting from the mutations.

**Genotyping**

Genotyping was performed on genomic DNA extracted from adult tail clips or whole larvae via PCR using oligos flanking the mutation site (oligo sequences in table 1) followed by agarose gel electrophoresis on a 2% agarose gel to identify mutant fragments. PCR reactions consist of the following: 5x MyTaq Buffer (Bioline) 5µL, forward primer (10 µM) 1µL, reverse primer (10 µM) 1 µL, MyTaq DNA polymerase (Bioline) 0.2 µL, nuclease free water (Ambion) 16.8 µL, DNA 1 µL. Reaction conditions: 95°C for 60s followed by 40 cycles of 95°C for 15s, annealing temperature (primer dependant) for 15s, 72°C for 10s before a final 72°C for 5 minutes.

**Light Microscopy**

All light microscopy was performed using a Leica MZ APO Stereo microscope with a Dino-lite AM7025X eyepiece camera for image acquisition. Images were processed using FIJI (Schindelin *et al.*, 2012).

**Spontaneous tail coiling assay.**

24 hpf embryos were recorded under a light microscope in groups of 15, for 1-minute intervals. Once recorded, embryos were placed into individual wells of a microplate and positions recorded. Tail flicks were counted manually upon rewatching the recorded videos. Only full rotations were recorded, ignoring minor twitches. Following on, embryos were culled, and DNA extracted for genotyping.

**Touch Response Assay**

A petri dish with E3 medium was placed onto a white LED backlight board with a clamp holding a mobile phone above recording at 60 frames per second. 48 hpf dechorionated embryos were placed into the centre of the petri dish and using a pipette tip, were tapped on the back of the head and reaction recorded until movement had fully stopped. If there was no reaction, then up to 3 more taps were performed until the fish was removed and discounted from the experiment. When an appropriate number had been recorded, samples were taken for genotyping. Total displacement, mean velocity and peak acceleration were quantified using FIJI software using the ‘TrackMate’ plug-in (Schindelin *et al.*, 2012). Peak acceleration was calculated from the first point at which velocity no longer increased after stimulation.

**Zebrafish morphology measurements**

Larvae were imaged as described. Scale was set by measuring an object of known distance (a ruler) and set in FIJI. Standard length (SL), snout – vent length (SVL), head-tail angle (HTA), eye area (EA) and swim bladder area (SBA) were all measured using the drawing tools in FIJI.

**Relative mtDNA copy number.**

Relative mtDNA copy number was measured using a protocol established previously (Rahn *et al.*, 2015). Primers targeting the mitochondrial gene NADH dehydrogenase-1 (*mt-nd1*) and a nuclear gene, elongation factor 1 (*elf1a*) or beta-2-microglobulin (*b2m*) were used (table 1). The relative mtDNA copy number was calculated as the ΔCt between nuclear gene and mitochondrial gene.

**Protein isolation and quantification.**

Protein was extracted on ice with RIPA buffer (Sigma-Aldrich) and protease inhibitor (cOmplete, mini EDTA free protease inhibitor cocktail, Roche). Manual homogenisation was carried out with Teflon pestles until no visible chunks of tissue were present. Lysed samples were incubated on ice for 30 minutes followed by a centrifugation step at 8000RPM for 10 minutes. Supernatants were transferred to fresh tubes for analysis or stored at -20°C. Protein concentration of lysates was quantified using a Pierce™ BCA Protein Assay Kit (ThermoFisher) following the manufacturer’s instructions and a FLUOstar Omega microplate reader.

**Immunoblotting**

Equal amounts of protein (between 10 – 20µg) was mixed with 7.5μL NuPage LDS sample buffer (4X), 3μL NuPAGE Sample Reducing Agent (10X) and the remaining volume to 30uL with deionised water. If mitochondrial proteins were of interest, the lysate mix was heater to 37°C for 10 minutes prior to loading, otherwise samples were heated to 95°C for 10 minutes prior to loading. Samples were loaded into NuPAGE 4 to 12% Bis-Tris mini protein gels (ThermoFisher) with a PageRuler Plus Prestained Protein Ladder (10 to 180kDa). Gels were run in NuPAGE MES SDS running buffer at 120V until the desired amount of separation had occurred. Proteins were transferred to a PVDF membrane using an iBlot 2 dry transfer device, subsequently membranes were blocked in 5% milk (TBS-T (0.1% Tween), skimmed milk powder) at room temperature for 1 hour. Primary antibodies were prepared in 5% milk to desired concentration and incubated with membranes overnight at 4°C. The following day, membranes were washed at least 3 times for periods of 15 minutes with TBS-T before incubation with a secondary HRP antibody targeting the species of the primary and incubated at RT for one hour. Membranes were washed for another 3 periods of 15 minutes with TBS-T (0.1% tween) before being developed. Blots were incubated with SuperSignal West Pico PLUS Chemiluminescent Substrate for 4 minutes, before being developed with an Amersham Imager 680. Blots were quantified in FIJI software. Primary antibodies used include anti-RRM2B (SAB1405069) and anti-alpha Tubulin (GTX124303),

**Deoxynucleoside supplementation**

Deoxyribonucleosides: 2-deoxyadenosine monohydrate (Sigma, D7400), 2'-deoxyguanosine monohydrate (Sigma, D7145), 2-deoxycytidine (Sigma, D3897) & thymidine (Sigma, T9250) were dissolved in either E3 or DMSO to a stock concentration of 100mM (200X) and frozen at -20°C. Adenosine deaminase inhibitor, EHNA hydrochloride (Sigma, E114), was dissolved in E3 or DMSO to a stock concentration of 10mM (200X) and frozen at -20°C. Experiments have shown that not co-supplementing with a deaminase inhibitor results in almost total loss of any supplemented 2'-deoxyguanosine monohydrate in cells (Blázquez-Bermejo *et al.*, 2019). Embryos were supplemented with deoxynucleosides at 50μM (1X) and ENHA hydrochloride at 5μM (1X) in a volume of 30mL of E3 medium. If deoxynucleosides dissolved in DMSO were used, control dishes were supplemented with DMSO to 0.5% concentration.

**Light/Dark Transition Test**

At 4 dpf, larvae were placed into a 96 well transparent microtiter plate with 200μL of E3 medium/treatment medium and kept overnight at 28°C to acclimatise to the plate. The following day, using a Zantiks MWP system (Zantiks Ltd), movement was recorded over an 80-minute period with alternating periods of 10 minutes light and 10 minutes dark. For quantification, only movement in the first 5 minutes of cycles 2-4 was used to allow for initial habituation to the stimuli and to avoids desensitisation to the stimuli.

**L-lactate measurement**

To measure L-lactate in 7 dpf larvae, 3 pools of 20 larvae were collected per treatment condition and following the manufacturers colorimetric protocol, L-lactate measured immediately using the L-Lactate Assay Kit (Abcam, ab65330) with the Deproteinizing Sample Preparation Kit – TCA (ab204708).

**Statistical Analysis**

All statistical analysis was performed in Graph Pad Prism v8.4.3. All data to be statistically analysed was first assessed for normality with a Shapiro-Wilk test. Further, if performing a comparison of two means, variance was assessed with an F-test. If multiple comparisons were being made, Brown-Forsythe test was performed. Based on results from these analyses, an appropriate statistical test was chosen. For comparison of two means, normally distributed data with equal variance an unpaired t-test was performed, otherwise a t-test with Welch’s correction was used for data with unequal variance or a nonparametric Mann-Whitney U test for comparison of data not normally distributed. For multiple comparisons, one-way ANOVA was used to compare means with normally distributed data and equal variance. For multiple comparisons with data of unequal variance, Welch’s ANOVA was used and a Kruskal-Wallis test for data not normally distributed. When performing multiple comparisons, to account for Type I error (false positives), post-hoc tests were performed, either Tukey post-hoc test (ANOVA) or Dunn’s multiple comparison (Kruskal-Wallis).

Results are considered significant with a p value/adjusted p value of >0.05. All behavioural and morphological data was collected and analysed blind, without knowing the genotype of the sample beforehand. For statistical analysis at least three biological replicates per sample type were taken as minimum. In the instances of immunoblotting or tissue deoxynucleoside quantification where there was limited tissue due to the necessity to pool larvae, which resulted in less than three biological replicates, no statistical test could be performed. To ensure technical accuracy of assays, a minimum of three technical replicates were performed per biological replicate.

The ‘light/dark transition test’ would occasionally track an non-larvae point (often light reflection) oscillating between larvae and the non-larvae point, giving the impression of very high activity for an individual larvae. This, when identified in the data, was validated from the video recording and then removed from the analysis using the ROUT method. The ROUT method is a statistical method that detects outliers from a data set based on the false discovery rate, given as Q, where in this instance Q was set at 1% as indicated by Moutulsky and Brown (Motulsky and Brown, 2006).

**Supplementary References**

Blázquez-Bermejo, C. *et al.* (2019) ‘Increased dNTP pools rescue mtDNA depletion in human POLG-deficient fibroblasts’, *FASEB Journal*, 33(6), pp. 7168–7179. Available at: https://doi.org/10.1096/fj.201801591R.

Hill, J.T. *et al.* (2014) ‘Poly peak parser: Method and software for identification of unknown indels using sanger sequencing of polymerase chain reaction products’, *Developmental Dynamics*, 243(12), pp. 1632–1636. Available at: https://doi.org/10.1002/dvdy.24183.

Moreno-Mateos, M.A. *et al.* (2015) ‘CRISPRscan: Designing highly efficient sgRNAs for CRISPR-Cas9 targeting in vivo’, *Nature Methods*, 12(10), pp. 982–988. Available at: https://doi.org/10.1038/nmeth.3543.

Motulsky, H.J. and Brown, R.E. (2006) ‘Detecting outliers when fitting data with nonlinear regression – a new method based on robust nonlinear regression and the false discovery rate’, *BMC Bioinformatics*, 7(1), p. 123. Available at: https://doi.org/10.1186/1471-2105-7-123.

Schindelin, J. *et al.* (2012) ‘Fiji: an open-source platform for biological-image analysis’, *Nature Methods*, 9(7), pp. 676–682. Available at: https://doi.org/10.1038/nmeth.2019.
